# Accuracy of malaria rapid diagnosis test Optimal-IT® in Kinshasa, the Democratic Republic of Congo

**DOI:** 10.1186/1475-2875-11-224

**Published:** 2012-07-06

**Authors:** Hypolite Mavoko Muhindo, Gillon Ilombe, Ruth Meya, Patrick M Mitashi, Albert Kutekemeni, Didier Gasigwa, Pascal Lutumba, Jean-Pierre Van Geertruyden

**Affiliations:** 1 Département de Médecine Tropicale, Université de Kinshasa, B.P. 747, Kin XI, République Démocratique du Congo; 2 Programme National de Lutte contre le Paludisme, Ministère de la Santé Publique, Kinshasa, République Démocratique du Congo; 3 International Health Unit, Department of Epidemiology, University of Antwerp, Campus Drie Eiken, Universiteitsplein 1, 2610, Wilrijk, Belgium

**Keywords:** Rapid Diagnostic Test, Malaria, Optimal-IT®, Paracheck-Pf®, Democratic Republic of Congo

## Abstract

**Background:**

Despite some problems related to accuracy and applicability, malaria rapid diagnostic tests (RDTs), are currently considered the best option in areas with limited laboratory services for improving case management and reducing over-treatment. However, their performance must be established taking into the account the particularities of each endemic area. In the Democratic Republic of Congo, the validity of Optimal-IT**®** and Paracheck-Pf®, respectively based on the detection of lactate dehydrogenase and histidine-rich protein-2, was assessed at primary health care level (PHC).

**Methods:**

This was a two-stage cluster randomized survey, conducted in one health centre in 12 health zones in Kinshasa city. All patients with malaria presumptive diagnosis were eligible. Gold standard was microscopy performed by experts from the parasitology unit, Kinshasa University.

**Results:**

624 patients were enrolled. 53.4% (95% CI: 49.4-57.3) owed a bed net, obtained in 74.5% of cases (95% CI: 69.4-79.1) through community-based distribution by the National Malaria Control Programme. Microscopy expert reading confirmed 123 malaria cases (19.7%; 95% CI: 16.7-23.1). Overall sensitivity were 79.7% (95% CI: 72.4-86.8), 87.8% (95% CI: 81.9-93.6) and 86.2% (95% CI: 79.9-92.3), respectively, for Optimal-IT®, Paracheck-Pf**®** and microscopy performed at PHC. Specificity was 97.0% (95% CI: 95.5-98.5), 91.6% (95% CI: 89.1-94.0) and 49.1% (95% CI: 44.7-53.4). The proportion of confirmed cases seemed similar in under-fives compared to others. Any treatment prior to the current visit was a predictor for malaria (AOR: 2.3; 95% CI: 1.5-3.5), but not malaria treatment (AOR: 0.87; 95% CI: 0.4-1.8). Bed net ownership tended to protect against malaria (AOR: 0.67; 95% CI: 0.45-0.99).

**Conclusion:**

Although microscopy is considered as the "gold standard" for malaria diagnosis at point of care level, this study showed that its accuracy may not always be satisfactory when performed in health centres.

## Background

Despite the efforts engaged in control, malaria remains a major concern for the public health, especially in sub-Saharan Africa. The hope to control malaria was hampered by spread of resistance to anti-malarial drugs. Irrational anti-malarial drug use contributed to the selection of drug resistant strains [1]. The spread of resistance to the cheap first-line treatment (chloroquine, sulphadoxine-pyrimetamine) led endemic countries to adopt efficacious and more expensive artemisinin-based combination threrapy (ACT). To delay the spread of resistance to ACT, the World Health Organization (WHO) recently recommended prompt parasitological confirmation prior to malaria treatment [2-4]. Parasitological confirmation is crucial because presumptive treatment based on clinical diagnosis, results in thousands of inappropriate treatments. This has not only economic consequences, but increases anti-malarial drug pressure and delays specific non-malaria treatment [5-7]. This policy should focus on the primary health care (PHC) level, where most uncomplicated malaria cases are managed. This implies that health centres (HC) should have accurate tools and human skills for reliable malaria diagnosis and that the recommended anti-malarial drugs should be available.

Microscopy is considered the gold standard for malaria diagnosis. However, this requires a well-maintained microscope, staining performed according to standard procedures and a good expertise in microscopy. These conditions are not always easy to meet in limited-resource HC [8]. Many authors agree that rapid diagnostic tests (RDTs) may be a good alternative where microscopy is not available [9-13]. Today, in Democratic Republic of Congo (DRC), malaria diagnosis relies on microscopy and more and more on RDTs. However, the accuracy of both methods should be absolutely assessed. The malaria control community does not always pay enough attention to the type of RDT used for malaria diagnosis [14], considering that they are over 100 different brands available. Most of them are based commonly on the detection of either histidine-rich protein 2 (HRP2), a protein synthesized by *Plasmodium falciparum,* lactate dehydrogenase (LDH) or aldolase, two enzymes produced by all human *Plasmodium* species.

Many studies have shown that HRP2 remains longer in circulation as LDH or aldolase following a successful malaria treatment [15-21]. This may increase the proportion of false positive results. Therefore, HRP2-based tests may not be ideal for areas with high malaria transmission because of repeated attacks. However, LDH disappears fast after successful treatment [8,18,22,23]. Therefore, LDH-based RDTs may be suitable in DRC, a context with stable malaria transmission.

The aim of this study was to assess, the validity of a LDH-based RDT (Optimal-IT**®)** and a HRP-2-based RDT (Paracheck-Pf®) at primary health care level in DRC.

## Methods

### Study setting and design

Data were collected from 9 May to 7 June 2011 in Kinshasa, the capital city of DRC. Kinshasa is the largest city, with around 10 million inhabitants. The climate is tropical, raining from October to May, but malaria transmission is permanent during the whole year. The actual entomological inoculation rate is not known, but data published 19 years ago reported up to 1,200 infecting bites/person/year [24].

This was a two-stage cluster randomized survey. For the first stage, 12 health zones out of 35 were randomly selected: Bandalungwa, Binza ozone, Bumbu, Kimbanseke, Kingabwa, Kokolo, Limete, Makala, Maluku 1, Masina 1, Police and Selembao. For the second stage, one private or public HC in each health zone was randomly selected among those reporting monthly to the National Health Information System. The rationale for the choice of this design was to allow at the same time the assessment of the PHC microscopy accuracy and to describe the malaria case management at PHC level in Kinshasa (Results and discussion concerning malaria case management will be reported in another manuscript).

Considering a 98% sensitivity of Optimal-IT®, a sample size of at least 264 patients was needed to find, with 90% power and 95% confidence, a sensitivity of at least 95%. Taking into the account a possible cluster effect, 624 patients were included in this survey.

Optimal-IT® (DiaMed Basel, Switzerland) was chosen for its good performance in areas of stable transmission [25,26] and the brevity of the LDH antigen [8]. Paracheck-Pf® (Orchid Biomedical Systems, Goa, India) is thought to be the most used RDT in DRC [27], but its accuracy in Eastern DRC was reported to be unsatisfactory with a low specificity of 52% [20]. The Optimal-IT® devices used had batch numbers OK0027M and OK0030M (expiration date 04/ 2012). For Paracheck-Pf® the batch number was 311030 (expiration date 11/ 2012). All devices were stored according to manufacturer requirements.

### Study procedures

Data collection in HCs was carried out by laboratory technicians who were members of the study team. They obtained informed consent, filled out a questionnaire, prepared blood slides and performed RDTs, under the supervision of researchers from the Tropical Medicine Department, Kinshasa University. The aim of the supervision was to ensure the compliance to the study procedures as well as to recheck RDT interpretation. A one-day training workshop was conducted on the study standards operating procedures (SOPs). The expert microscopists were senior laboratory technicians with over fifteen years of work/research experience at the Kinshasa University

All male and female outpatients attending study centres with clinical suspicion of malaria and to whom a blood smear was asked for confirmation, were eligible for inclusion in the survey. Lack of informed consent constituted the exclusion criteria. Those who gave consent were consecutively enrolled. Medical history, ownership and use of bed nets, socio-demographic data and attitudes before and during the illness course were recorded on CRFs.

Blood for thick/ thin smears and RDTs were collected from the same finger prick and prepared on the same slide bearing the patient’s identification code. About 5 μl of blood was drawn by the study team using a loop provided with the RDT device. The test preparation and interpretation was done following manufacturer’s instructions. The tests were considered positive when the antigen and control lines were visible in their respective windows, negative when only the control band was visible and invalid when the control band was not visible. In case of invalid result, the RDT was done once again.

Blood smears were stained with 10% Giemsa for 10 minutes. Thin smears were fixed with methanol prior to staining. Slides prepared by the study team were first examined by the HC laboratory technicians, blinded to RDTs results and using the WHO semi-quantitative method [28]. Their results were recorded on CRFs. All slides were stored in secured slide-boxes and read by two expert microscopists at the Parasitology Unit, Tropical Medicine Department, University of Kinshasa. The expert microscopists were blinded to HC microscopy and RDT results. In case of >15% discrepancy, a senior laboratory technician’s judgement was required. The parasite density was estimated assuming 8,000 white blood cells/μl [28] and the final result was the mean parasite density. The thin smear was used for species identification.

### Statistical analysis

Data were double-entered and validated in Epi info version 3.5.1 software and analysed using Stata version 11 (Stata Corp, Lakeway, College Station, Texas, USA). The Sensitivity, Specificity, PPV and NPV of the history of fever (presumptive treatment), HC microscopy, Optimal-IT® and Paracheck-Pf® were determined with expert microscopy as gold standard.

### Ethical approval

Ethical approval for this study was provided by Ethical committees of the University Hospital, Antwerp, Belgium and the School of Public Health, Kinshasa University, DRC. Before inclusion, written informed consent was obtained from participants/ legal guardians for minors.

## Results

### Socio-demographic profile and baseline characteristics

**Figure 1:**
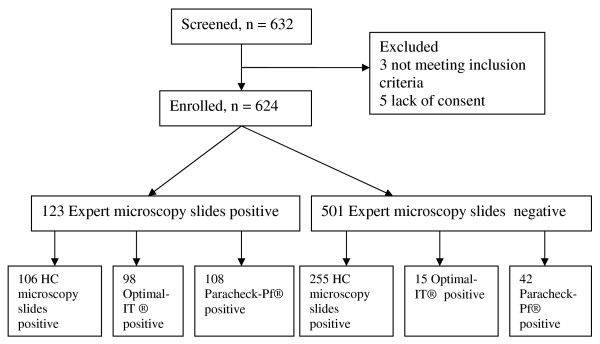
Study profile.

From 9 May to 7 June 2011, 632 patients were screened. Eight were excluded: three for not meeting the inclusion criteria and five who refused to consent (Figure 1). In the 624 included, the median age was 22 years (range: 0.03-87), 136 (21.8%) were under five years of age, 362 (58%) were female and 373 (59.8%) reported a history of fever. Three hundred and thirty three (53.4%) owned a bed net, of whom 248 (74.5%) were obtained through community-based distribution by the National Malaria Control Programme (NMCP). Among those owing a bed net, 224(67.3%) slept under it the night before enrolment. Those who did not use it gave following reasons: heat (44.0%), felt uncomfortable (26.6%), forgot (3.7%). Other reasons (25.7%) were: out last night, net dilapidated, no mosquitoes, the net causes some diseases. One hundred and forty four (23.1%) had an insect screen on the house windows, of which 62.5% were in a good condition. 335 (53.6%) had used medications before enrolment, of whom 47 (14.0%) included anti-malarial.

Expert microscopists detected 123 malaria positive slides of whom 120 were *P. falciparum*, two were *P. falciparum* mixed with *Plasmodium ovale* and one was *Plasmodium malariae* (Table 1). The median parasitaemia was 19,490/μl (range: 66-736,940). At the HC, 361 slides were positive (57.9%), whereas Optimal-IT® gave positive results in 113 (18.1%) and Paracheck-Pf® in 150 (24.0%) patients. Invalid results were rare. Stratified by age category (under-fives compared to others), the difference of positive cases detected by each technique was not significant (Table 1). Any treatment prior to the current visit was a predictor for malaria (AOR: 2.3; 95% CI:1.5-3.5), but not malaria treatment (AOR: 0.87; 95% CI:0.4-1.8). Bed net ownership tended to protect against malaria (AOR: 0.67; 95% CI:0.45-0.99).

**Table 1:** Overall results of diagnostic techniques

**Variable**	**All age group**		**< 5 years**		**>5 years**		**p**
	**n/N**	**% (95%CI)**	**n**	**%(95%CI)**	**n**	**%(95%CI)**	
Optimal-IT® positive	113/624	18.1 (15.2–21.4)	32	23.5(16.3-30.7)	81	16.6(13.2-19.9)	0.063
Paracheck-Pf ® positive	150/624	24.04 (20.7-7.4)	36	26.5(19.0-34.0)	114	23.4(19.6-27.1)	0.453
Health centre microscopy slides positive	361/624	57.9 (53.9–61.8)	84	61.8(53.5-70.0)	277	56.7(52.3-61.1)	0.296
Expert microscopy slide positive*	123/624	19.7 (16.7 – 23.1)	33	24.3(17.0-31.5)	90	18.4(15.0-21.9)	0.131

*Three cases were *P. malariae* and mixed infection (*P. falciparum* + *P. ovale*).

### Accuracy of diagnostic methods assessed

Considering the expert microscopy as the gold standard, the sensitivity of the presumptive treatment, HC microscopy, Optimal-IT® and Paracheck-Pf® was respectively 82.9% (95% CI: 76.1-89.6), 86.2% (95% CI: 79.9-92.3), 79.7% (95% CI: 72.4-86.8) and 87.8% (95% CI: 81.9-93.6). Overall, no technique reached the positive predictive value (PPV) of 90%, whereas the negative predictive value (NPV) was over 90% for all cases (Table 2). Considering the parasitaemia >100/ μl, the sensitivity of Optimal-IT® and Paracheck-Pf® was respectively 82.0% (95%CI: 75.5-89.0) and 90.6% (95%CI: 85.3-96.0). For parasitaemia range of 100 to 1,000/μl, the sensitivity of Optimal-IT® was much lower. However, both RDTs had almost a same sensitivity when parasitaemia was above 1,000/μl (Table 3).

**Table 2:** Overall sensitivity, specificity, PPV and NPV of malaria diagnostic methods with expert microscopy as gold standard, stratified by age categories

	**Sensitivity % (95% CI)**	**Specificity % (95% CI)**	**PPV % (95% CI)**	**NPV % (95% CI)**
**All age groups**				
History of fever	82.9 (76.1-89.6)	45.9 (41.5-50.2)	27.3 (22.8-31.8)	91.6 (88.2-95.0)
Health centre microscopy	86.2 (79.9-92.3)	49.1 (44.7-53.4)	29.4 (24.6-34.0)	93.5 (90.5-96.5)
Optimal-IT®	79.7 (72.4-86.8)	97.0 (95.5-98.5)	86.7 (80.4-93.0)	95.1 (93.2-97.0)
Paracheck-Pf®	87.8 (81.9-93.6)	91.6 (89.1-94.0)	72.0 (64.7-79.2)	96.8 (95.2-98.4)
**Patients < 5 years**				
History of fever	97.0 (90.7-100)	11.7 (5.3-19.9)	26.0 (18.1-33.8)	92.3 (77.0-100)
Health centre microscopy	90.9 (80.5-100)	47.6 (37.7-57.4)	35.7 (25.3-46.1)	94.2 (87.8-100)
Optimal-IT®	87.9 (76.1-99.6)	97.0 (93.7-100)	90.6 (80.2-100)	96.2 (92.4-100)
Paracheck-Pf®	90.9 (80.5-101.2)	94.2 (89.6-98.7)	83.3 (70.9-95.8)	97.0 (93.6-100)
**Patients>5 years**				
History of fever	77.8 (69.0-86.5)	54.8 (49.8-59.7)	28.0 (22.4-33.6)	91.6 (88.0-95.1)
Health centre microscopy	84.4 (76.8-92.0)	49.5 (44.5-54.4)	27.4 (22.1-32.7)	93.4 (90.0-96.7)
Optimal-IT®	76.7 (67.7-85.5)	97.0 (95.2-98.6)	85.2 (77.3-93.0)	94.8 (92.7-97.0)
Paracheck-Pf®	86.7 (79.5-93.8)	91.0 (88.1-93.7)	68.4 (59.8-77.0)	96.8 (95.0-98.6)

PPV = Positive Predictive Value, NPV = Negative Predictive Value, 95% CI = 95% Confidence Interval.

**Table 3:** Sensitivity of OptiMAL-IT® and Paracheck-Pf® at different levels of parasitaemia

**Microscopy parasitaemia ranges**	**n**	**Optimal-IT® positive**	**Sensitivity (%)**	**Paracheck-Pf® positive**	**Sensitivity (%)**
< 100	3	2	66.7	2	66.7
100 – 1,000	19	9	47.3	16	84.2
> 1,000	98	87	88.8	90	91.8

## Discussion

In this study, the sensitivity of Optimal-IT® did not reach the acceptable threshold of 90% [29], whereas the specificity was 97%. In fact, in this setting, none of both RDTs, Paracheck-Pf® and Optimal-IT®, reached the sensitivity threshold of 95% for parasitaemia>100/ μl recommended by WHO [30]. Some authors reported over 90% sensitivity and specificity of Optimal-IT® [13,25,26,31]. With sensitivity and specificity of respectively 100% and 94% in Tanzania, Tarimo *et al*[25] even commented that Optimal-IT® accuracy was almost ideal. The poorer performance observed in this study may be related to the batch-to-batch variation or failure to maintain the cold chain. Such variation has been reported in a comprehensive study of the WHO [13]. In Eastern Congo, Swarthout *et al*[20] reported sensitivity and specificity of respectively 100% and 52% for Paracheck-Pf® in children under-fives, indicating that almost the half of the sick without malaria were considered positive. However, some RDT positive test may have submicroscopic parasite load and be wrongly considered as false positive because expert microscopy is conventionnally considered as gold standard on point of care level. In the present study, a higher specificity of 94.2% was found in the same age group. Nevertheless the sensitivity was lower than what Swarthout reported. Four years ago, the NMCP assessed the accuracy of Paracheck-Pf® in four sites in DRC (Kinshasa, Lubumbashi, Goma and Kisangani), the overall sensitivity and specifity (88.0% and 94.4%) were comparable to what are reported in this study (unpublished data). In this study, the sensitivity of Paracheck-Pf® was higher than Optimal-IT® for parasitaemia range of 100-1,000/ μl (Table 3). This confirms former reports that indicated that LDH based RDTs were less sensitive than HRP2 based ones. [13]. However, this statement could not be extended to the category of parasitaemia < 100/ μl due to the small number (3 cases).

Although many authors agree that RDTs should be only considered where microscopy is not available [9-13], this logic may be questionable. This survey revealed that routine microscopy in health facilities is far from being considered as gold standard for malaria diagnosis at PHC level in DRC. Indeed, with a PPV of 29.4%, one should realize that two third of the detected malaria cases are false positive, received unneeded anti-malarial drugs and hence were not treated for any other disease. The low specificity observed in this study can be attributed to limitation of light microscopy as described by Payne [32]. But the main explanation might be the poor quality of malaria microscopists in DRC. Such results have already been reported by other authors [9,27,33,34]. To overcome these limitations, new health policies that highlight the importance of strengthening laboratory services are needed, as well asadequate funding, and the implementation of a quality assurance system [35]. The last should ensure that:

(i) there is constant training, supervision of staff and quality control of their tasks;

(ii) the structure of the programme is practical and sustainable with adequate staff and resources;

(iii) slide collection, staining and reading are accurate, timely and linked to clinical diagnosis;

(iv) results are quickly provided to clinicians;

(v) clinicians can trust the results; and

(vi) there is logistic support to provide quality supplies and equipment.

The training may be the backbone of the improvement. In Uganda, refresher training in malaria microscopy has shown a substantially impact [36]. Such experience should be planned in DRC. Another option would be, according to Shillcutt *et al*[12], to prefer RDTs to microscopy. Although both RDTs evaluated did not reach the sensitivity threshold of 95% recommended by WHO for parasitaemia > 100/ μl [30], taking into account the poor quality of microscopy observed, we may consider to rely on RDT for malaria diagnosis on PHC level. However, a further issue would be to assess, in an evidence-based manner, which RDT from the hundreds brands available on the market, is most suitable in the DRC context? The deployment of RDTs countrywide is not easy due to the weak governance. The NMCP included RDTs as part of malaria diagnostic two years ago, but recently, during a three-day workshop for evaluation it was noticed that very little progress had been made towards this goal. A possible reason of the slow deployment could be the current international financial context. In fact, the health system in DRC is mostly supported by outside partners, so the success of a strategy depends on the support provided. The recent NMCP workshop highlighted the importance of an evaluation of the RDT needs for the next five years, involving all traditional funding agencies to quantify their expected contribution, so that the government could take care of the gap. Currently, in the DRC, RDTs are procured by the Global Funds, World Bank and some NGOs. Patients do not pay the real price of RDT which is for some tests as high as 1 USD. The decision to change from an RDT to another or to combine or not with microscopy should take into account the efficiency of these alternatives in terms of cost-effectiveness and could include price of RDT, cost of re-training, effectiveness of RDT versus effective and excellent microscopy, cost of good performing of microscopy (training, quality assurance), cost of materials, reagents and equipement.

The parasite load in HC was not assessed as all HC laboratory technicians used the semi-quantitative scale expressed from + to ++++ according to numbers of asexual parasites per high-power field [28], whereas study expert readers expressed the parasite density as asexual parasites/μl. This latter method was introduced by the NMCP only one year ago, and this might be the reason why it is not yet common at PHC level. The fever could have been considered instead of history of fever, but body temperature was not systematically recorded. In many cases, the nurse just appreciates subjectively the body temperature by touching the patient, without using a thermometer. This is an issue to be addressed during refresher training of nurses at PHC.

Presumptive treatment based on history of fever appears to be the worst diagnostic method of all assessed, especially in children under five years old. Indeed, the low specificity of 11.7% in area of low malaria endemicity created a situation where three-quarters of the presumed malaria cases had no malaria (PPV = 27.3%). Therefore, this practice has to be discouraged in order to reduce unneeded prescription of ACT, reduce anti-malarial drug pressure and decrease delay of specific non-malaria treatment.

Malaria in patients presenting at health centre level was low as only 19.7% of presumptive malaria cases were confirmed by expert microscopy. This may be the result of several interventions, including ACT availability and community-based distribution of mosquito nets. However, the impact of these interventions on malaria transmission is not documented in DRC. In fact, in Kinshasa, more recent data published 19 years ago reported an entomological inoculation rate of up to 1,200 infecting bites/man/year [24]. It is likely that significant change has occurred since 1993. Fifty three percent of the population owned a mosquito net. The coverage was consistent with the one observed in Mozambique [37], but much is still to be done in order to comply with WHO requirement of achieving a full coverage of population at risk of malaria. Three-quarters of the bed nets were provided through community-based distribution organized over the past 5 years by the NMCP and two third of the bed net owners slept under mosquito net the previous night. These findings are encouraging, compared to outside Kinshasa two years ago and which reported less than half of the participants sleeping under a net [38]. The relatively high utilization may be attributable to a sensibilization of the population. However, this is also not documented. Contrary to the study of Ndjinga and Minakwa [38], the mosquito net usage was not different between age groups (p = 0.175). Bed net ownership tended to protect against malaria (AOR: 0.67; 95% CI: 0.45-0.99). This is consistent with findings reported by Atieli *et al*[39] in the highlands of western Kenya. It is likely that the level of protection might increase with the level of coverage and usage.

## Conclusion

Although microscopy is considered as the "gold standard" for malaria diagnosis at point of care level, this study showed that its accuracy may not always be satisfactory when performed in health centres. This suggests an urgent need for capacity building of microscopists and/or the implementation of accurate RDT on a large scale. Once this has been achieved, the next step and also the cornerstone to maintain the accuracy of malaria diagnosis will be the implementation/ strengthening of the quality control system.

## List of abbreviations

ACT: Artemisinin-based Combination Therapy; DRC: Democratic Republic of Congo; HC: Health Centre; NGO: Non Govermental Organization; NMCP: National Malaria Control Programme; PHC: Primary Health Care; RDT: Rapid Diagnostic Test; WHO: World Health Organization.

## Competing interests

Authors declare that they have no conflict of interest.

## Authors’ contribution

HMM, PL and JPV designed the study protocol. HMM, RM, PM and GI supervised activities on the field. HMM performed the statistical analysis. HMM, PL and JPV wrote the first draft of the manuscript. All authors read and approved the final version.
